# Factors associated with diagnostic stage of hip osteoarthritis due to acetabular dysplasia among Japanese female patients: a cross-sectional study

**DOI:** 10.1186/s12891-016-1179-4

**Published:** 2016-08-02

**Authors:** Satoko Ohfuji, Seiya Jingushi, Kyoko Kondo, Muroto Sofue, Moritoshi Itoman, Tadami Matsumoto, Yoshiki Hamada, Hiroyuki Shindo, Yoshio Takatori, Harumoto Yamada, Yuji Yasunaga, Hiroshi Ito, Satoshi Mori, Ichiro Owan, Genji Fujii, Hirotsugu Ohashi, Shinji Takahashi, Yoshio Hirota

**Affiliations:** 1 Department of Public Health, Graduate School of Medicine, Osaka City University, 1-4-3, Asahi-machi, Abeno-ku, Osaka, 545-8585 Japan; 2 Department of Orthopaedic Surgery, Kyushu Rosai Hospital of Japan Labor Health and Welfare Organization, Kitakyushu, Japan; 3 Orthopaedic Division, Nakajo Central Hospital, Niigata, Japan; 4 Department of Orthopedic Surgery, School of Medicine, Kitasato University, Sagamihara, Japan; 5 Hakutokai Takao Hospital, Hachioji, Japan; 6 Department of Orthopaedic Surgery, Kanazawa Medical University, Uchinada, Japan; 7 Orthopaedic Division, Mitsuwadai General Hospital, Chiba, Japan; 8 Department of Orthopaedic Surgery, Graduate School of Medicine, Nagasaki University, Nagasaki, Japan; 9 Department of Orthopaedic Surgery, Japan Community Health Care Organization Yugawara Hospital, Kanagawa, Japan; 10 Department of Orthopaedic Surgery, Fujita Health University School of Medicine, Toyoake, Japan; 11 Department of Orthopaedic Surgery, Hiroshima Prefectural Rehabilitation Center, Hiroshima, Japan; 12 Department of Orthopaedic Surgery, Asahikawa Medical University, Asahikawa, Japan; 13 Department of Bone and Joint Surgery, Seirei Hamamatsu General Hospital, Hamamatsu, Japan; 14 Department of Orthopaedic Surgery, Okinawa Red Cross Hospital, Naha, Japan; 15 Tohoku Hip Joint Center, Matuda Hospital, Sendai, Japan; 16 Department of Orthopedic Surgery, Saiseikai Nakatsu Hospital, Osaka, Japan; 17 Department of Orthopaedic Surgery, Graduate School of Medicine, Osaka City University, Osaka, Japan; 18 College of Healthcare Management, Miyama, Japan

**Keywords:** Anthropometric factors, Body weight, Disease severity, Epidemiology, Hip osteoarthritis

## Abstract

**Background:**

In Japan, the majority of hip osteoarthritis (OA) was caused by acetabular dysplasia, and about 90 % of patients were female. The present study focused on Japanese female patients with hip OA due to acetabular dysplasia, and examined the associated factors with OA staging at diagnosis, in special reference to body weight.

**Methods:**

Study subjects were 336 Japanese women who were newly diagnosed with hip OA caused by acetabular dysplasia at 15 hospitals in 2008. The self-administered questionnaire elicited patients’ body weight at age 20 and at OA diagnosis. Four ranked OA staging according to radiographic findings of the hip joint (pre-OA, initial stage, advanced stage or terminal stage) was regarded as the outcome index. Proportional odds models in logistic regression were used to calculate odds ratios (ORs) and 95 % confidence intervals (CIs) for severer stage of OA.

**Results:**

At diagnosis, 45 % of patients suffered from terminal stage of OA, whereas 13 % and 14 % were categorized into pre-OA and initial stage, respectively. After adjustment for potential confounders, weight gain since age 20 revealed the increased ORs for severer OA stage at diagnosis (OR 2.02; 95 % CI, 1.07–3.80). Other significant characteristics were age (67+ vs. 20–49 years, OR 12.4), lower education (junior high school vs. junior college or higher, OR 4.00), parity (OR 2.19), lower acetabular head index (<60.0 vs. 71.1+, OR 2.36), and longer duration since symptom onset (6.0+ vs. <1.0 year, OR 2.94).

**Conclusions:**

Weight gain since age 20 might be involved in mechanisms of OA development, which is independent of age or severity of acetabular dysplasia.

## Background

Hip osteoarthritis (OA) is one of the leading causes of disability in older adults. Once people develop the disease, they feel difficulty walking, which decreases their quality of life [1]. This problem has been increasing with the current ageing of the population. Thus, it is necessary to identify factors to control disease development.

Obesity is thought to contribute to the development and progression of OA through increased mechanical load and, hormonal and metabolic mechanisms [2, 3]. To the best of our knowledge, two systematic reviews [4, 5] and one meta-analysis [6] have identified obesity as a risk factor for the development of hip OA. However, a number of studies also reported no association between body mass index (BMI) and hip OA [7–11]. Even in studies that reported an increased risk of hip OA among subjects with higher BMI, the magnitude of the association between BMI and hip OA seemed to be relatively lower than that between BMI and knee OA [12–14].

These results were mainly derived from Western countries, where the aetiology of hip OA is mostly primary OA. To date, no previous study have reported the association between obesity and hip OA in Japan, where the aetiology and background characteristics of hip OA are different from Western countries. In Japan, the majority of hip OA was caused by acetabular dysplasia, whereas only about 10 % of patients were primary OA. Besides, female patients accounted for about 90 % [15, 16]. We therefore targeted Japanese female patients with secondary hip OA due to acetabular dysplasia, and examined the associated factors with OA staging at diagnosis, in special reference to body weight. The present study focused on the weight after age 20, when complete closure of the growth plate in the hips has usually occurred, and investigated the association of weight at age 20, the current weight, and weight change on the risk of severer disease stage at diagnosis.

## Methods

### Subjects

A multi-institutional epidemiological study was conducted for patients newly diagnosed with hip OA at 15 hospitals in five areas of Japan: four hospitals in the Kyushu and Okinawa areas, two hospitals in the Shikoku, Chugoku and Kinki areas, three hospitals in the Chubu areas, three hospitals in the Kanto areas, and three hospitals in the Tohoku and Hokkaido areas. The patients were limited to those whose hip joints had completed closure of the growth plate. Exclusion criteria was those who had been operated on both hip joints after growth plate closure. Hip OA was defined as a symptomatic hip joint that had patients’ complains of hip pain and had radiological evidence of OA (i.e., joint space narrowing, sclerotic changes of the subchondral bone, osteophyte formation, or a bone cyst). A symptomatic hip joint with a deformity in the joint, such as acetabular dysplasia or dislocation, but no osteoarthritic changes was also included. The study was approved by the institutional review board in each participating hospital.

From January to September 2008, a total of 485 newly diagnosed hip OA patients were recruited: 158 patients (33 %) were recruited from four hospitals in the Kyushu and Okinawa areas, 98 patients (20 %) were recruited from two hospitals in the Shikoku, Chugoku and Kinki areas, 101 patients (22 %) were recruited from three hospitals in the Chubu areas, 109 patients (22 %) were recruited from three hospitals in the Kanto areas, and 19 patients were recruited from three hospitals in the Tohoku and Hokkaido areas. Among those, 390 patients (81 %) had a hip OA due to acetabular dysplasia. Of these 390 patients, 358 patients (92 %) were female and aged 20 years or more, and were identified as candidates for the present study. All patients provided written informed consent after the nature and possible consequences of the study had been explained.

### Information collection

At the time of recruitment, the following information was obtained from patients using a self-administered questionnaire: current age (years); age at hip pain onset (years); weight at age 20 and current age (kg); comorbidity including stroke, hypertension, heart disease, liver disease, diabetes mellitus, liver disease, pulmonary disease, etc. (none or present); smoking (never, past or current); alcohol drinking (never, past or current); sports club activity at school (none or present); years of education (junior high school, high school or junior college or higher); occupation (physical worker or not); and parity (none or present).

In addition, each subject’s orthopedist completed a structured questionnaire to collect clinical information regarding: measured height (cm) and weight (kg) at OA diagnosis; the acetabular dysplasia indices based on radiographic evaluation; and disease stage at OA diagnosis (as a study outcome) [15, 16]. The following acetabular dysplasia indices were chosen according to the reproducibility for the multi-institutional examination [17] from the Sharp angle [18], the center edge angle [19], the acetabular roof obliquity angle [20], the acetabular head index (AHI) [21], and the approximate acetabular quotient [21]: Sharp angle, acetabular roof obliquity angle, and AHI which were measured in an antero-posterior view radiograph of the bilateral hip joints.

As a study outcome, the disease stage of hip OA was classified into one of the four stages (i.e., pre-OA stage, initial stage, advanced stage, and terminal stage) according to the guidelines proposed by the Japanese Orthopedic Association’s committee [22]. Briefly, pre-OA stage was regarded as a symptomatic hip joint which demonstrated no radiological OA changes but showed morphological changes of the acetabulum and/or proximal femur related to OA. The joints at the initial stage were that one or more OA changes with possible narrowing of the joint space were appeared, in addition to that the width of the joint space was maintained at 2 mm or more throughout the weight-bearing area. The joints at the advanced stage were that the width of the joint space was less than 2 mm at the thinnest point or that loss of the joint space was less than 15 mm. The joints at the terminal stage showed gross loss of the joint space which expanded to 15 mm or more. The assessment of radiographic grade of OA was performed by a single orthopedist at each collaborating hospital.

### Statistical analysis

For main exposure variables, body weights at age 20 and current age were categorized into quartile according to the distribution of patients. In addition, weight change since age 20 were calculated and categorized into three levels (more than 2 kg decreased; almost no change: ±2 kg; and more than 2 kg increased). BMI at age 20 and current age were categorized into three levels (<18.5/ 18.5–24.9/ 25.0+ kg/m^2^), according to the WHO classification, and BMI change since age 20 was categorized into three levels (more than 1.5 kg/m^2^ decreased; almost no change: ±1.5 kg/m^2^; and more than 1.5 kg/m^2^). Regarding other explanatory variables, continuous variables were categorized into quartile except for indices of acetabular dysplasia (i.e., Sharp angle, acetabular roof obliquity angle, and AHI). Since data for indices of acetabular dysplasia included a relatively larger number of missing data, these variables were categorized into tertile.

To examine the association with disease stage at OA diagnosis, proportional odds models in logistic regression were used to calculate odds ratios (ORs) and 95 % confidence intervals (CIs). The trend of association was assessed by assigning ordinal numbers to the increasing exposure categories. Kruskal-Wallis test, Wilcoxon rank sum test or the Chi-square test were also used where appropriate. Variables that showed a p-value of less than 0.10 in age-adjusted model or that seemed to medically relate to the outcome were considered as potential confounders for adjustment in the final model.

In addition, to consider the potential interaction between current age, weight change since age 20, duration since hip pain onset and OA stage at diagnosis, stratified analysis by age was also conducted. All analyses were performed with SAS Ver 9.1 (SAS Institute, Inc., Cary, NC, USA).

## Results

Of 358 newly diagnosed female hip OA patients with acetabular dysplasia, 22 did not respond the self-administered questionnaire. Eventually, 336 comprised the subjects for the analysis. Table 1 shows the background characteristics of these 336 patients. Median age at diagnosis was 57.7 years (range: 23.2–84.5 years). Median weight and BMI increased as age increased. About half of the patients had any comorbidities. As for the clinical characteristics, about 60 % of the patients suffered from bilateral OA. Radiographic indices of acetabular dysplasia showed median values of 46.0 for the Sharp angle, 23.0 for the acetabular roof obliquity angle, and 65.5 for the AHI. Median duration between hip pain onset and OA diagnosis was 2.0 years. Among the 336 patients, 45 % of patients suffered from terminal stage of OA, whereas 13 % and 14 % were categorized into pre-OA and initial stage, respectively.

**Table 1:** Characteristics of Japanese women with newly diagnosed hip osteoarthritis caused by acetabular dysplasia^a^

Characteristics			n (%) or median (range)
Age (years)		(*n* = 336)	57.7 (23.2–84.5)
Height (cm)		(*n* = 336)	153 (135–173)
Weight at age 20 (kg)		(*n* = 320)	50 (36–85)
Weight at diagnosis (kg)		(*n* = 336)	53 (31–105)
Weight change between age 20 and diagnosis (kg)		(*n* = 320)	4 (−28–40)
Body mass index at age 20 (kg/m^2^)		(*n* = 320)	20.9 (15.8–35.8)
Body mass index at diagnosis (kg/m^2^)		(*n* = 336)	22.8 (15.8–43.1)
Body mass index change between age 20 and diagnosis (kg/m^2^)		(*n* = 320)	1.6 (−13–16.7)
Presence of comorbidity	Present	(*n* = 335)	157 (47)
Smoking	Never	(*n* = 334)	265 (79)
	Past		36 (11)
	Current		33 (10)
Consumption of alcohol	Never	(*n* = 333)	202 (61)
	Past		15 (5)
	Current		116 (35)
Sports club activities at school	Present	(*n* = 334)	155 (46)
Education	Junior high school	(*n* = 333)	46 (14)
	High school		186 (56)
	Junior college or higher		101 (30)
Physical work	Present	(*n* = 331)	27 (8)
Parity	Present	(*n* = 334)	269 (81)
Clinical information			
Involved side	Hemilateral	(*n* = 336)	141 (42)
	Bilateral		195 (58)
Radiographic index of acetabular dysplasia	Sharp angle	(*n* = 255)	46 (27–58)
	Acetabular roof obliquity angle	(*n* = 252)	23 (0–53)
	Acetabular head index (%)	(*n* = 253)	65.5 (35.7–105.7)
Duration since symptom onset (years)		(*n* = 330)	2.0 (0–43.0)
Disease stage at OA diagnosis	Pre-OA	(*n* = 336)	43 (13)
	Initial stage		48 (14)
	Advanced stage		95 (28)
	Terminal stage		150 (45)

^a^Variables are expressed as no. (%) or median (range)

Table 2 shows the associations between selected characteristics and disease stage at the time of OA diagnosis. On univariate analysis, significantly increased ORs were observed for higher age, weight change since age 20, BMI change since age 20, presence of comorbidity, lower education, parity, higher acetabular roof obliquity angle, lower AHI, longer duration since symptom onset, whereas significantly decreased ORs were obtained for taller height, past smokers, current drinkers, and sports club activities at school. However, when considered the effect of age, the association with BMI change since age 20, presence of comorbidity, sports club activities at school vanished.

**Table 2:** Crude and age-adjusted association between selected characteristics and disease severity at diagnosis

Characteristics	Disease stage at OA diagnosis, n (%)				Univariate	Age-adjusted
	Pre OA	Initial	Advanced	Terminal	OR (95 % CI)	OR (95 % CI)
Age (years)						
20–49	30 (70)	24 (50)	20 (21)	16 (11)	1.00	
50–57	5 (12)	11 (23)	37 (39)	29 (19)	4.26 (2.42–7.51)	
58–66	7 (16)	8 (17)	18 (19)	45 (30)	7.83 (4.29–14.3)	
67+	1 (2)	5 (10)	20 (21)	60 (40)	15.1 (8.02–28.4)	
					(Trend *P* < 0.01)	
Height (cm)						
<150.0	4 (9)	4 (8)	22 (23)	46 (31)	1.00	1.00
150.0–153.9	5 (12)	14 (29)	29 (31)	48 (32)	0.65 (0.36–1.17)	0.67 (0.37–1.24)
154.0–157.9	18 (42)	9 (19)	20 (21)	27 (18)	0.30 (0.16–0.55)	0.46 (0.24–0.88)
158.0+	16 (37)	21 (44)	24 (25)	29 (19)	0.27 (0.15–0.49)	0.53 (0.28–0.99)
					(Trend *P* < 0.01)	(Trend *P* = 0.04)
Weight at age 20 (kg)						
<46.0	7 (17)	12 (26)	23 (25)	39 (28)	1.00	1.00
46.0–49.9	6 (14)	11 (24)	25 (27)	32 (23)	0.89 (0.50–1.61)	0.89 (0.48–1.63)
50.0–54.9	15 (36)	14 (30)	25 (27)	35 (25)	0.65 (0.38–1.14)	0.68 (0.38–1.22)
55.0+	14 (33)	9 (20)	18 (20)	35 (25)	0.78 (0.44–1.40)	0.93 (0.51–1.71)
					(Trend *P* = 0.24)	(Trend *P* = 0.59)
Weight change since age 20 (kg)						
<−2	10 (24)	7 (15)	11 (12)	34 (24)	2.14 (1.15–3.96)	1.87 (0.98–3.55)
±2	16 (38)	14 (30)	23 (25)	26 (18)	1.00	1.00
>2	16 (38)	25 (54)	57 (63)	81 (57)	1.86 (1.15–3.02)	1.80 (1.08–2.98)
					(Trend *P* = 0.71)	(Trend *P* = 0.56)
Weight at diagnosis (kg)						
<48.5	11 (26)	10 (21)	25 (26)	38 (25)	1.00	1.00
48.5–53.4	10 (23)	16 (33)	19 (20)	43 (29)	1.04 (0.60–1.82)	0.94 (0.53–1.67)
53.5–60.4	14 (33)	15 (31)	17 (18)	35 (23)	0.78 (0.44–1.36)	0.74 (0.41–1.34)
60.4+	8 (19)	7 (15)	34 (36)	34 (23)	1.02 (0.58–1.79)	1.38 (0.76–2.48)
					(Trend *P* = 0.83)	(Trend *P* = 0.43)
Body mass index at age 20 (kg/m^2^)						
<18.5	4 (10)	10 (22)	13 (14)	12 (9)	0.64 (0.35–1.19)	0.77 (0.41–1.45)
18.5–24.9	35 (83)	32 (70)	71 (78)	117 (83)	1.00	1.00
25.0+	3 (7)	4 (9)	7 (8)	12 (9)	1.02 (0.48–2.15)	1.16 (0.53–2.54)
					(Trend *P* = 0.23)	(Trend *P* = 0.36)
Body mass index change since age 20 (kg/m^2^)						
<−1.5	8 (19)	6 (13)	9 (10)	25 (18)	1.59 (0.85–2.97)	1.04 (0.54–2.01)
±1.5	22 (52)	18 (39)	27 (30)	44 (31)	1.00	1.00
>1.5	12 (29)	22 (48)	55 (60)	72 (51)	1.59 (1.02–2.47)	1.34 (0.84–2.14)
					(Trend *P* = 0.41)	(Trend *P* = 0.26)
Body mass index at diagnosis (kg/m^2^)						
<18.5	3 (7)	4 (8)	5 (5)	7 (5)	0.74 (0.32–1.72)	1.25 (0.51–3.03)
18.5–24.9	34 (79)	37 (77)	56 (59)	105 (70)	1.00	1.00
25.0+	6 (14)	7 (15)	34 (36)	38 (25)	1.30 (0.82–2.07)	1.45 (0.89–2.37)
					(Trend *P* = 0.16)	(Trend *P* = 0.28)
Presence of comorbidity						
None	27 (63)	32 (67)	49 (52)	70 (47)	1.00	1.00
Present	16 (37)	16 (33)	45 (48)	80 (53)	1.69 (1.13–2.52)	0.87 (0.56–1.36)
Smoking						
Never	30 (70)	31 (65)	82 (88)	122 (81)	1.00	1.00
Past	9 (21)	9 (19)	7 (8)	11 (7)	0.40 (0.21–0.75)	0.46 (0.24–0.89)
Current	4 (9)	8 (17)	4 (4)	17 (11)	0.94 (0.48–1.84)	1.94 (0.94–3.99)
					(Trend *P* = 0.20)	(Trend *P* = 0.53)
Consumption of alcohol						
Never	19 (44)	16 (33)	63 (68)	104 (70)	1.00	1.00
Past	2 (5)	4 (8)	4 (4)	5 (3)	0.45 (0.17–1.15)	0.58 (0.22–1.57)
Current	22 (51)	28 (58)	26 (28)	40 (27)	0.41 (0.26–0.62)	0.60 (0.39–0.94)
					(Trend *P* < 0.01)	(Trend *P* = 0.02)
Sports club activities at school						
No	16 (38)	21 (44)	52 (55)	90 (60)	1.00	1.00
Present	26 (62)	27 (56)	42 (45)	60 (40)	0.57 (0.38–0.85)	0.83 (0.54–1.26)
Education						
Junior high school	3 (7)	1 (2)	9 (10)	33 (22)	1.00	1.00
High school	19 (44)	30 (64)	59 (63)	78 (52)	1.45 (0.93–2.25)	1.18 (0.75–1.87)
Junior college or higher	21 (49)	16 (34)	26 (28)	38 (26)	4.83 (2.32–10.0)	2.39 (1.08–5.31)
					(Trend *P* < 0.01)	(Trend *P* = 0.06)
Physical work						
No	40 (95)	43 (93)	86 (91)	135 (91)	1.00	1.00
Present	2 (5)	3 (7)	8 (9)	14 (9)	1.44 (0.68–3.04)	0.85 (0.39–1.85)
Parity						
None	19 (44)	10 (21)	12 (13)	24 (16)	1.00	1.00
Present	24 (56)	38 (79)	82 (87)	125 (84)	2.23 (1.36–3.66)	1.57 (0.93–2.65)
Clinical information						
Involved side						
Hemilateral	18 (42)	17 (35)	37 (39)	69 (46)	1.00	1.00
Bilateral	25 (58)	31 (65)	58 (61)	81 (54)	0.79 (0.53–1.17)	1.14 (0.75–1.75)
Sharp angle						
<45	13 (30)	11 (28)	30 (38)	39 (42)	1.00	1.00
45–48	20 (47)	14 (35)	23 (29)	30 (32)	0.62 (0.36–1.06)	1.04 (0.59–1.84)
49+	10 (23)	15 (38)	26 (33)	24 (26)	0.72 (0.41–1.25)	1.55 (0.85–2.83)
					(Trend *P* = 0.22)	(Trend *P* = 0.16)
Acetabular roof obliquity angle						
<20	25 (58)	15 (38)	16 (20)	29 (33)	1.00	1.00
20–26	13 (30)	14 (35)	33 (42)	23 (26)	1.34 (0.78–2.32)	1.11 (0.63–1.95)
27+	5 (12)	11 (28)	30 (38)	37 (42)	2.61 (1.49–4.57)	2.77 (1.54–5.00)
					(Trend *P* < 0.01)	(Trend *P* < 0.01)
Acetabular head index (%)						
71.1+	21 (50)	15 (38)	22 (28)	27 (29)	1.00	1.00
60.0–71.0	13 (31)	14 (35)	26 (33)	34 (37)	1.59 (0.92–2.73)	2.03 (1.15–3.59)
<60.0	8 (19)	11 (28)	31 (39)	31 (34)	1.83 (1.05–3.18)	2.98 (1.64–5.40)
					(Trend *P* = 0.03)	(Trend *P* < 0.01)
Duration since symptom onset (years)						
<1.0	17 (41)	16 (35)	19 (20)	28 (19)	1.00	1.00
1.0–1.9	13 (32)	8 (17)	23 (24)	31 (21)	1.50 (0.85–2.66)	1.53 (0.85–2.77)
2.0–5.9	5 (12)	13 (28)	36 (38)	41 (28)	1.99 (1.15–3.44)	2.08 (1.18–3.68)
6.0+	6 (15)	9 (20)	17 (18)	48 (32)	3.16 (1.75–5.70)	3.43 (1.85–6.34)
					(Trend *P* < 0.01)	(Trend *P* < 0.01)

After adjusted for remaining potential confounders (Table 3), patients with higher age had an increased OR for severe stage of the disease (Trend *P* < 0.01). In addition, weight gain since age 20 also showed about two-fold increased OR for severer stage of OA at diagnosis (OR 2.02; 95%CI, 1.07–3.80). Other significant characteristics were lower education (junior high school vs. junior college or higher, OR 4.00), parity (OR 2.19), lower AHI (<60.0 vs. 71.1+, OR 2.36), and longer duration since symptom onset (6.0+ vs. <1.0 year, OR 2.94). When weight at diagnosis was replaced with weight at age 20 and weight change since age 20 (Model 2), heavier weight was associated with severe stage of OA at diagnosis, with a marginally significance. In these models, use of the proportional odds model would be appropriate, because p values for the score to test for the proportional odds assumption were 0.32 in Model 1 and 0.08 in Model 2.

**Table 3:** Multivariate analysis about the association between selected characteristics and disease severity at diagnosis

Characteristics		Model 1^a^	Model 2^b^
		OR (95 % CI)	OR (95 % CI)
Age (years)	20–49	1.00	1.00
	50–57	3.32 (1.61–6.87)	3.31 (1.61–6.79)
	58–66	4.67 (2.15–10.2)	4.72 (2.19–10.1)
	67+	12.4 (5.31–28.7)	14.6 (6.32–33.7)
		(Trend *P* < 0.01)	(Trend *P* < 0.01)
Height (cm)	<150.0	1.00	1.00
	150.0–153.9	0.86 (0.38–1.93)	0.93 (0.44–2.00)
	154.0–157.9	0.74 (0.31–1.80)	0.76 (0.32–1.77)
	158.0+	0.69 (0.28–1.70)	0.70 (0.30–1.64)
		(Trend *P* = 0.40)	(Trend *P* = 0.35)
Weight at age 20 (kg)	<46.0	1.00	
	46.0–49.9	1.11 (0.51–2.40)	
	50.0–54.9	0.77 (0.36–1.65)	
	55.0+	1.34 (0.57–3.14)	
		(Trend *P* = 0.76)	
Weight change since age 20 (kg)	<−2	1.67 (0.74–3.81)	
	±2	1.00	
	>2	2.02 (1.07–3.80)	
		(Trend *P* = 0.27)	
Weight at diagnosis (kg)	<48.5		1.00
	48.5–53.4		1.02 (0.49–2.13)
	53.5–60.4		0.87 (0.41–1.85)
	60.4+		2.07 (0.97–4.41)
			(Trend *P* = 0.09)
Smoking	Never	1.00	1.00
	Past	0.64 (0.28–1.50)	0.63 (0.28–1.45)
	Current	1.57 (0.61–4.06)	1.68 (0.65–4.31)
		(Trend *P* = 0.68)	(Trend *P* = 0.60)
Consumption of alcohol	Never	1.00	1.00
	Past	0.61 (0.18–2.03)	0.52 (0.16–1.65)
	Current	0.70 (0.39–1.25)	0.66 (0.37–1.18)
		(Trend *P* = 0.22)	(Trend *P* = 0.15)
Education	Junior high school	1.00	1.00
	High school	1.32 (0.72–2.41)	1.24 (0.69–2.24)
	Junior college or higher	4.00 (1.49–10.7)	3.12 (1.22–8.02)
		(Trend *P* = 0.02)	(Trend *P* = 0.04)
Parity	None	1.00	1.00
	Present	2.19 (1.09–4.39)	2.43 (1.23–4.80)
Clinical information			
Acetabular head index (%)	71.1+	1.00	1.00
	60.0–71.0	1.44 (0.76–2.72)	1.83 (0.99–3.41)
	<60.0	2.36 (1.23–4.53)	2.43 (1.29–4.59)
		(Trend *P* < 0.01)	(Trend *P* < 0.01)
Duration since symptom onset (years)	<1.0	1.00	1.00
	1.0–1.9	1.34 (0.64–2.80)	1.37 (0.67–2.82)
	2.0–5.9	2.38 (1.18–4.81)	2.25 (1.14–4.44)
	6.0+	2.94 (1.33–6.48)	3.05 (1.42–6.57)
		(Trend *P* < 0.01)	(Trend *P* < 0.01)

^a^Model 1 included age, height, weight at age 20, weight change since age 20, smoking, consumption of alcohol, education, parity, acetabular head index, and duration since symptom onset (*N* = 234)

^b^Model 2 included age, height, weight at diagnosis, smoking, consumption of alcohol, education, parity, acetabular head index, and duration since symptom onset (*N* = 244)

Of the 336 subjects, 234 completed the data for the multivariate analysis (perfect responders), but 102 were missing some data (imperfect responders) (Table 4). Missing data was relatively frequent for radiographic indices of acetabular dysplasia, because subjects with terminal stage of OA were likely to break the hip joint and thus could not measure these angles radiographically in some subjects. Because of this potential bias, the imperfect responders, as compared to perfect responders, were older, experienced longer duration since symptom onset, and were diagnosed as a severer stage of OA. Second, we examined correlations between these three variables. The correlation coefficients with disease stage at diagnosis were: 0.49 (*P* < 0.01) for age, and 0.20 (*P* < 0.01) for duration since symptom onset.

**Table 4:** Comparison of selected characteristics between perfect and imperfect responders

Characteristics		No. of missing data	n (%) or median (range)		*P*
			Perfect responders (*N* = 234)	Imperfect responders (*N* = 102)	
Age (years)		0	56.0 (23.2–84.5)	59.3 (24.1–82.0)	0.02
Height (cm)		0	154 (138–173)	153 (135–172)	0.09
Weight at age 20 (kg)		16	50 (36–85)	48 (37–70)	0.30
Weight change since age 20 (kg)		16	4 (−28–40)	3.5 (−14–35)	0.51
Weight at diagnosis (kg)		0	54 (31–88)	52 (35–105)	0.02
Body mass index at age 20 (kg/m^2^)		16	20.9 (16.4–35.8)	20.8 (15.8–28.8)	0.64
Body mass index change since age 20 (kg/m^2^)		16	1.6 (−13.0–16.7)	1.4 (−6.3–14.3)	0.50
Body mass index at diagnosis (kg/m^2^)		0	22.9 (15.8–38.1)	22.05 (17.8–43.1)	0.18
Smoking	Never	2	188 (80)	77 (77)	0.69
	Past		25 (11)	11 (11)	
	Current		21 (9)	12 (12)	
Consumption of alcohol	Never	3	141 (60)	61 (62)	0.95
	Past		11 (5)	4 (4)	
	Current		82 (35)	34 (34)	
Education	Junior high school	3	32 (14)	14 (14)	0.95
	High school		132 (56)	54 (55)	
	Junior college or higher		70 (30)	31 (31)	
Parity	Present	2	189 (81)	80 (80)	0.87
Clinical information					
Involved side	Hemilateral	0	100 (43)	41 (40)	0.66
	Bilateral		134 (57)	61 (60)	
Radiographic index of acetabular dysplasia					
	Sharp angle	81	46 (27–58)	45 (39–54)	0.81
	Acetabular roof obliquity angle	84	23 (0–53)	25 (12.5–43.0)	0.48
	Acetabular head index (%)	83	65.6 (35.7–105.7)	65 (40.9–85.7)	0.77
Duration since symptom onset (years)		6	1.5 (0–40.0)	2.0 (0–43.0)	<0.01
Disease stage at OA diagnosis					
	Pre-OA	0	39 (17)	4 (4)	<0.01
	Initial stage		37 (16)	11 (11)	
	Advanced stage		77 (33)	18 (18)	
	Terminal stage		81 (35)	69 (68)	

*P value was calculated by Wilcoxon rank sum test for continuous variables or the Chi-square test for categorical variables

Next, to examine the interactive effect of age at OA diagnosis, weight gain since age 20 and duration from symptom onset for disease stage at OA diagnosis, stratified analysis by age was conducted (Table 5). As a result, the positive association with weight gain since age 20 was observed with a marginal significance among patients who were diagnosed with hip OA in younger age (i.e., 20–57 years), but the association with duration from symptom onset was not observed among those aged group. On the other hand, among patients diagnosed with hip OA in the older age (i.e., 58 years or more), both heavier weight at age 20 and heavier weight at diagnosis were associated with severe stage of hip OA, and these association retained with marginal significance. In addition, longer duration from symptom onset was also associated with severer OA in older aged patients.

**Table 5:** Association between selected characteristics and disease severity, according to age at diagnosis

Characteristics		20–57 years	58 years or more
		OR (95 % CI) P	OR (95 % CI) P
Weight at 20 years of age (kg) ^a^	<46.0	1.00	1.00
	46.0–49.9	1.87 (0.63–5.54) 0.26	0.56 (0.17–1.92) 0.36
	50.0–54.9	0.89 (0.30–2.69) 0.84	0.74 (0.22–2.50) 0.62
	55.0+	1.18 (0.39–3.60) 0.78	4.43 (0.76–26.0) 0.09
		(Trend *P* = 0.86)	(Trend *P* = 0.26)
Weight change since age 20 (kg) ^a^	<−2	1.86 (0.67–5.16) 0.23	0.48 (0.09–2.61) 0.40
	±2	1.00	1.00
	2+	1.98 (0.89–4.41) 0.09	1.05 (0.34–3.27) 0.93
		(Trend *P* = 0.54)	(Trend *P* = 0.38)
Weight at diagnosis (kg) ^b^	<48.5	1.00	1.00
	48.5–53.4	0.79 (0.29–2.16) 0.64	1.80 (0.56–5.86) 0.33
	53.5–60.4	0.98 (0.38–2.58) 0.97	1.07 (0.32–3.64) 0.91
	60.4+	1.55 (0.59–4.06) 0.37	3.71 (0.93–14.7) 0.06
		(Trend *P* = 0.33)	(Trend *P* = 0.15)
Duration from symptom onset (years)^a^	<1.0	1.00	1.00
	1.0–1.9	1.30 (0.47–3.62) 0.62	2.21 (0.61–8.09) 0.23
	2.0–5.9	2.22 (0.85–5.79) 0.10	3.95 (1.16–13.5) 0.03
	6.0+	1.64 (0.57–4.76) 0.36	14.3 (3.08–66.5) <0.01
		(Trend *P* = 0.20)	(Trend *P* < 0.01)

^a^Model included height, weight at age 20, weight change since age 20, smoking, consumption of alcohol, education, parity, acetabular head index, and duration since symptom onset (*N* = 234)

^b^Model included height, weight at diagnosis, smoking, consumption of alcohol, education, parity, acetabular head index, and duration since symptom onset (*N* = 244)

## Discussion

Present study indicated that weight gain since age 20 was associated with disease severity at OA diagnosis, particularly in patients who were diagnosed with hip OA due to acetabular dysplasia in less than 58 years of age. On the other hand, among those diagnosed hip OA in the older age, lasting heavier weight since age 20 rather than weight gain since age 20 was highly affected for disease stage at OA diagnosis. These results suggest that, for subjects with acetabular dysplasia, both weight gain in early adult life and heavier weight lasting for the long time might be involved in mechanisms of OA development. To date, no study have investigated the association between weight change and hip OA development among subjects with acetabular dysplasia. However, a possible explanation for the association with weight gain in early adult life or heavier weight lasting for the long time is that such patients’ hip joints suffered from a high load for a long time. Besides, when considering the fact that the development of hip OA caused by acetabular dysplasia results from the joint subluxation, weight gain in early adult life (when people’s activities would be increased) might bring about the more load to the joints. Taken these possible mechanisms together, the present results seemed to be reasonable.

In the present study, other harmful characteristics for disease stage at OA diagnosis were older age, lower education, parity, lower AHI, and longer duration since symptom onset. Hip OA is a common form of arthritis in the elderly [1]. Age has been shown to be a potential OA risk factor in previous studies [23, 24]. As for the lower education, previous study also indicated the lower education level was at increased risk for hip OA [25]. Subjects with lower education are likely to have unskilled or physical work, and therefore might be more likely to develop hip OA. Although our study failed to detect the significant association of physical work with disease stage at OA diagnosis, other Japanese study showed that physical work would be an important risk factor against hip OA [26]. Parity has been pointed out as the potential risk factor for hip OA [27]. One prospective cohort study indicated that the risk of hip replacement increased with increasing parity [27]. Since each pregnancy results in a period of increased body weight, the relationship between parity and hip OA might be explained by the increased body weight during pregnancy. In the present study, while lower AHI was used as an indicator of severer acetabular dysplasia, lower AHI showed the higher OR for severe OA stage at OA diagnosis. There are some cohort studies reported acetabular dysplasia as a risk factor of hip OA [28, 29]. In addition, recent Korean study showed that the association with acetabular dysplasia was more obviously observed when criteria of radiographic hip OA was more strictly defined [30]. Since the present study used the severity of OA as a study outcome, present results seemed to be corresponded to the Korean study. Besides, it could be considered a reasonable result that subjects who experienced longer duration since symptom onset were likely to have severer disease stage at OA diagnosis, based on the natural history of the disease in general. The results suggested that subjects with hip pain should visit the hospital as soon as possible before the disease progresses.

However, the present study had the following limitations. First, since self-reported body weights at age 20 was used in the assessment, they may be inaccurate compared with the measured values. However, a longitudinal study confirmed that correlation between recalled weights at ages 18 when participants were 50 years old and measured weights at ages 18 was 0.87 [31]. In addition, some inaccuracy, if any, was considered to be non-differential, since all of the study subjects suffered from the same disease, hip OA due to acetabular dysplasia. This non-differential misclassification could only lead to underestimating the association, but it would not invalidate the present results. Second, potential bias due to imperfect responders in multivariate model might affect the present results. When we considered the potential bias in Table 4, it can be interpreted that imperfect responders included the severer stage of OA, and thus they were older and experienced longer duration since symptom onset. Since the analyzed study subjects in multivariate model biased the milder stage of OA than the total OA subjects, this potential bias might lead to the underestimation of the association with severe stage of OA. Third, we could not suggest the detailed timing of weight gain, since the detailed information on the weight gain could not be obtained. Weight gain can be affected by systemic illnesses, pregnancy, and diseases of lower limbs etc. Thus, further studies are needed to examine the effect of these conditions.

## Conclusion

The present study showed that weight gain since age 20 or lasting heavier weight since age 20 was associated with severe stage of OA at diagnosis among Japanese female subjects with acetabular dysplasia. Thus, weight control in early adult life is particularly important in preventing the development of hip OA.

## Abbreviations

AHI, acetabular head index; BMI, body mass index; CI, confidence interval; OA, osteoarthritis; OR, odds ratio
